# Editorial: The Role of Different Subcellular Organelles in DNA Damage Response

**DOI:** 10.3389/fcell.2021.760023

**Published:** 2021-10-28

**Authors:** Guoping Zhao

**Affiliations:** Key Laboratory of High Magnetic Field and Ion Beam Physical Biology, Anhui Province Key Laboratory of Environmental Toxicology and Pollution Control Technology, Hefei Institutes of Physical Science, Chinese Academy of Sciences, Hefei, China

**Keywords:** DNA damage repair, subcellular organelles, mitochondria, lysosomes, crosstalk

Cellular responses to DNA damage are important determinants of both cancer development and the outcome following radiation therapy and chemotherapy. The relationship between endonuclear repair proteins and DNA damage repair pathways during cancer genesis and treatment has been studied widely. However, the interaction between extra-nuclear subcellular organelles and DNA damage response has rarely been investigated. In this section, a comprehensive understanding of the role of different subcellular organelles in response to DNA damage and the possible mechanisms are described. This research provides new insights into cancer treatment with extra-nuclear subcellular organelles.

Mitochondria are considered to be one of the most important subcellular organelles that respond to various stressors, including DNA damage. Previous reports have indicated that exogenous threat-induced double strand breaks (DSBs) may be mediated by the production of mitochondrial reactive oxygen species (Ziech et al., 2011). Moreover, DNA damage repair is an energy-dependent process, and cellular energy depletion always leads to incomplete repair and cell death (Lans et al., 2012). Mitochondria regulate DNA damage repair pathways not only through the production of mitochondrial metabolites in the tricarboxylic acid cycle but also through mitochondria–nuclear signaling related to apoptosis and mitophagy. Han et al. proposed a key role for cytochrome C, a proapoptotic factor residing in mitochondria, in regulating DNA damage response (DDR) and radiosensitivity upon ultra-high dose rate (FLASH) radiation. Kim et al. described a comprehensive overview of mitophagy in the DNA damage response, which highlights a key mechanism for the induction of DDR through mitophagy. Similar to nuclear DNA, mitochondrial DNA (mtDNA) is also constantly assaulted by both exogenous and endogenous stresses. Rong et al. presented state-of-the-art knowledge on the role of base excision repair (BER), direct reversal (DR), mismatch repair (MMR), and double-strand break repair (DSBR) in the processing of the mtDNA repair system, which provides insights into the development of novel treatment strategies. In plants, chloroplasts are semiautonomous organelles with self-contained genetic materials (cp-DNA). Owing to its proximity with the reactive oxygen species (ROS)-generating electron transport system, the chloroplast genome is threatened by diverse forms of oxidative damages. Mahapatra et al. reviewed the existence of highly efficient repair machinery for DNA damage in chloroplasts, which has become an emerging field of research for better understanding of plant cell functions under stressed conditions.

Lysosomes are not only known as organelles that degrade waste materials but also serve as signaling hubs that regulate various metabolic pathways. Several results indicate that lysosomes are related to the DNA damage response, and lysosome-targeting therapy can increase radiosensitivity (Zhang et al., 2021). Lysosomes were reported to play a critical role in mediating mTOR activation and, inhibiting mTOR impairs the recruitment of DNA repair proteins, BRCA1 and RAD51, to DNA repair foci, leading to the suppression of DNA damage repair (Zhang et al., 2018). Moreover, TFEB, which has recently been identified as a major regulator of lysosomal biogenesis, is also involved in the removal of oxidative stress and γH2AX formation (Brady et al., 2018). Yang et al. reported that through limited activation of lysosome-mediated autophagy, cancer stem cells achieve increased survival. Lysosomes also regulate the radio-resistance of tumor cells through hypoxia. Zhang et al. demonstrated that hypoxia is the most important factor contributing to radio-resistance. Hypoxia also affects the distribution of lysosomes and decreases mTOR activity. Thus, we assume that hypoxia-induced radio-resistance is lysosome-dependent. However, additional studies are required to verify this hypothesis.

Several studies have reported that DNA damage triggers Golgi fragmentation. This response requires the Golgi protein GOLPH3 to link the Golgi and DNA damage. Upon DNA damage, DNA-PK phosphorylation of GOLPH3 increases its binding to MYO18A, which consequently reduces trafficking and enhances the survival of cancer cells (Farber-Katz et al., 2014). Xie et al. demonstrated the significance of another Golgi protein, SPCA1, in the regulation of DNA repair. The increased SPCA1 level lowered the intracellular Ca^2+^ level, probably by pumping Ca^2+^ into the Golgi, leading to a reduction in ROS level, eventually decreasing MAPK activity and diminishing senescence after radiation. Crosstalk between DDR and ribosomes and DDR and endoplasmic reticulum (ER) has recently been reported. In general, autophagic recycling of ribosomes and the ER is one of the most representative events known to occur in response to DNA damage. Specific regulation of autophagy of ribosomes is mediated by the ubiquitin carboxyl terminal hydrolase Ubp3/Bre5 complex (Yin et al., 2020). DNA-damaging reagents induce the activity of the Ubp3/Bre5 complex, supporting the significance of ribophagy in DDR. Additionally, when ER stress is induced by DNA damage, IRE1a, PERK, and ATF6 play a role in the response to ER stress by interacting with DNA damage repair proteins, resulting in the formation of autophagosomes (Dufey et al., 2020). Kim et al. summarized the latest research that connects ribophagy and ER-phagy with defects in the DNA repair system and highlighted the importance of autophagy activated by DDR and appropriate regulation of autophagic organelles, suggesting insights for future studies.

Overall, this editorial described emerging relationships between the extra-nuclear subcellular organelles and DDR, which greatly contributes to further understanding of the regulatory networks and the potential applications of extra-nuclear subcellular organelles in DDR.

## Author Contributions

GZ wrote the manuscript, contributed to the article, and approved the submitted version.

## Funding

This work was supported by the National Natural Science Foundation of China (31870845 and 11835014), the Dean's Fund of Hefei Institute of Physical Science (YZJJZX202014), and the CAS Pioneer Hundred Talents Program (GZ).

## Conflict of Interest

The author declares that the research was conducted in the absence of any commercial or financial relationships that could be construed as a potential conflict of interest.

## Publisher's Note

All claims expressed in this article are solely those of the authors and do not necessarily represent those of their affiliated organizations, or those of the publisher, the editors and the reviewers. Any product that may be evaluated in this article, or claim that may be made by its manufacturer, is not guaranteed or endorsed by the publisher.

## References

[B1] Brady O. A. Jeong E. Martina J. A. Pirooznia M. Tunc I. Puertollano R. (2018). The transcription factors TFE3 and TFEB amplify p53 dependent transcriptional programs in response to DNA damage. Elife 7:40856. 10.7554/eLife.40856 30520728 PMC6292694

[B2] Dufey E. Bravo-San Pedro J. M. Eggers C. Gonzalez-Quiroz M. Urra H. Sagredo A. I.. (2020). Genotoxic stress triggers the activation of IRE1alpha-dependent RNA decay to modulate the DNA damage response. Nat. Commun. 11:2401. 10.1038/s41467-020-15694-y 32409639 PMC7224204

[B3] Farber-Katz S. E. Dippold H. C. Buschman M. D. Peterman M. C. Xing M. Noakes C. J.. (2014). DNA damage triggers Golgi dispersal via DNA-PK and GOLPH3. Cell 156, 413–427. 10.1016/j.cell.2013.12.023 24485452 PMC4018323

[B4] Lans H. Marteijn J. A. Vermeulen W. (2012). ATP-dependent chromatin remodeling in the DNA-damage response. Epigenet. Chromatin. 5:4. 10.1186/1756-8935-5-4 22289628 PMC3275488

[B5] Yin Z. Popelka H. Lei Y. Yang Y. Klionsky D. J. (2020). The roles of ubiquitin in mediating autophagy. Cells 9:2025. 10.3390/cells9092025 32887506 PMC7564124

[B6] Zhang X. Wang J. Li X. Wang D. (2018). Lysosomes contribute to radioresistance in cancer. Cancer Lett. 439, 39–46. 10.1016/j.canlet.2018.08.029 30217567

[B7] Zhang Z. Yue P. Lu T. Wang Y. Wei Y. Wei X. (2021). Role of lysosomes in physiological activities, diseases, and therapy. J. Hematol. Oncol. 14:79. 10.1186/s13045-021-01087-1 33990205 PMC8120021

[B8] Ziech D. Franco R. Pappa A. Panayiotidis M. I. (2011). Reactive oxygen species (ROS)–induced genetic and epigenetic alterations in human carcinogenesis. Mutat. Res. 711, 167–173. 10.1016/j.mrfmmm.2011.02.015 21419141

